# Adolescent exposure to Δ^9^-tetrahydrocannabinol alters the transcriptional trajectory and dendritic architecture of prefrontal pyramidal neurons

**DOI:** 10.1038/s41380-018-0243-x

**Published:** 2018-10-03

**Authors:** Michael L. Miller, Benjamin Chadwick, Dara L. Dickstein, Immanuel Purushothaman, Gabor Egervari, Tanni Rahman, Chloe Tessereau, Patrick R. Hof, Panos Roussos, Li Shen, Mark G. Baxter, Yasmin L. Hurd

**Affiliations:** 1 Fishberg Department of Neuroscience, New York, NY USA; 2 Department of Psychiatry, New York, NY USA; 3 Graduate School of Biological Sciences, New York, NY USA; 4 Addiction Institute of Mount Sinai, Friedman Brain Institute, New York, NY USA; 5 Department of Genetics and Genomic Sciences, Institute for Genomics and Multiscale Biology, — all at the Icahn School of Medicine at Mount Sinai, New York, NY USA; 6 Present Address: Department of Pathology and Cell Biology, Vagelos College of Physicians and Surgeons, Columbia University, New York, NY, USA

**Keywords:** Neuroscience, Addiction, Schizophrenia

## Abstract

Neuronal circuits within the prefrontal cortex (PFC) mediate higher cognitive functions and emotional regulation that are disrupted in psychiatric disorders. The PFC undergoes significant maturation during adolescence, a period when cannabis use in humans has been linked to subsequent vulnerability to psychiatric disorders such as addiction and schizophrenia. Here, we investigated in a rat model the effects of adolescent exposure to Δ^9^-tetrahydrocannabinol (THC), a psychoactive component of cannabis, on the morphological architecture and transcriptional profile of layer III pyramidal neurons—using cell type- and layer-specific high-resolution microscopy, laser capture microdissection and next-generation RNA-sequencing. The results confirmed known normal expansions in basal dendritic arborization and dendritic spine pruning during the transition from late adolescence to early adulthood that were accompanied by differential expression of gene networks associated with neurodevelopment in control animals. In contrast, THC exposure disrupted the normal developmental process by inducing premature pruning of dendritic spines and allostatic atrophy of dendritic arborization in early adulthood. Surprisingly, there was minimal overlap of the developmental transcriptomes between THC- and vehicle-exposed rats. THC altered functional gene networks related to cell morphogenesis, dendritic development, and cytoskeleton organization. Marked developmental network disturbances were evident for epigenetic regulators with enhanced co-expression of chromatin- and dendrite-related genes in THC-treated animals. Dysregulated PFC co-expression networks common to both the THC-treated animals and patients with schizophrenia were enriched for cytoskeletal and neurite development. Overall, adolescent THC exposure altered the morphological and transcriptional trajectory of PFC pyramidal neurons, which could enhance vulnerability to psychiatric disorders.

## Introduction

Cannabis is now more widely used than cigarettes among adolescents [1], which raises concern given that exposure to exogenous cannabinoids is linked to changes in adult neurobiology and behavior relevant to psychiatric disease, specifically psychotic disorders such as schizophrenia [1–3]. The psychoactive effects of *Cannabis sativa* are mediated predominately by Δ^9^-tetrahydrocannabinol (THC) binding to the cannabinoid 1 receptor (CB1R), a central component of the endocannabinoid (eCB) system [4]. Disturbances of CB1R signaling in the prefrontal cortex (PFC) are implicated in schizophrenia and THC administration acutely worsens core symptoms in patients with stable disease [5, 6]. The PFC continues to develop throughout adolescence [7–9] and dynamic fluctuations in components of the eCB system occur throughout adolescent development[10–12]. Considering the role of the eCB system in neuronal development and synaptic plasticity [13], it is likely that this controlled regulation relates to the fine-tuning of PFC circuits established during adolescence. As such, adolescent cannabis exposure likely induces long-term consequences by interfering with the architecture of PFC neurocircuitry. Indeed, human neuroimaging studies demonstrate that individuals with a history of adolescent cannabis use exhibit altered PFC volume [14–16] and function [17, 18], but the cellular and molecular phenotype of such disturbances remains unknown.

The prelimbic (PrL) subregion of the rodent ventromedial PFC mediates cognitive function, decision-making, and emotional regulation making it a central component of mesolimbic and cortical circuitries [19–22], the disruption of which is implicated in the etiology of multiple psychiatric illnesses including schizophrenia. Layer III of the PrL cortex is a key node of this circuitry as it is directly connected with the amygdala (particularly the basolateral nucleus), contralateral cortex, mediodorsal thalamus, and nucleus accumbens [21, 23, 24]. Pyramidal neurons in the PrL exhibit the most pronounced developmental pruning and the highest rate of spine turnover of PFC subdivisions during adolescence [25]. These neurons, which comprise ~ 80% of cortical neurons, express CB1R and are directly innervated by interneurons with abundant CB1Rs that together orchestrate the activity of cortical function [26, 27]. Despite the relative paucity of somatodendritic CB1R expression in layer II and III PrL pyramidal neurons, these cells are directly regulated by the eCB system [28, 29]. The dendritic architecture of pyramidal neurons is often used as a correlate for neuronal processing, but knowledge is lacking regarding the molecular phenotype of these cells or the long-term impact of THC exposure on their molecular development.

We directly measured the dendritic structure and molecular phenotype of layer III PrL pyramidal neurons in a rodent model of adolescent THC exposure known to reliably alter reward and addiction-related behavior in adulthood [30, 31]. This goal was accomplished through direct structural analyses and the development of a novel multidisciplinary approach, using laser capture microdissection (LCM) to isolate mRNA from discrete cortical cellular populations in a region- and layer-specific manner, suitable for next-generation sequencing with low RNA input. This strategy revealed unique long-term morphological and transcriptomic aberrations during development associated with adolescent THC exposure.

## Materials and methods

Detailed information for all material and methods is provided in the Supplemental Information.

### Rat THC model

**Fig. 1:**
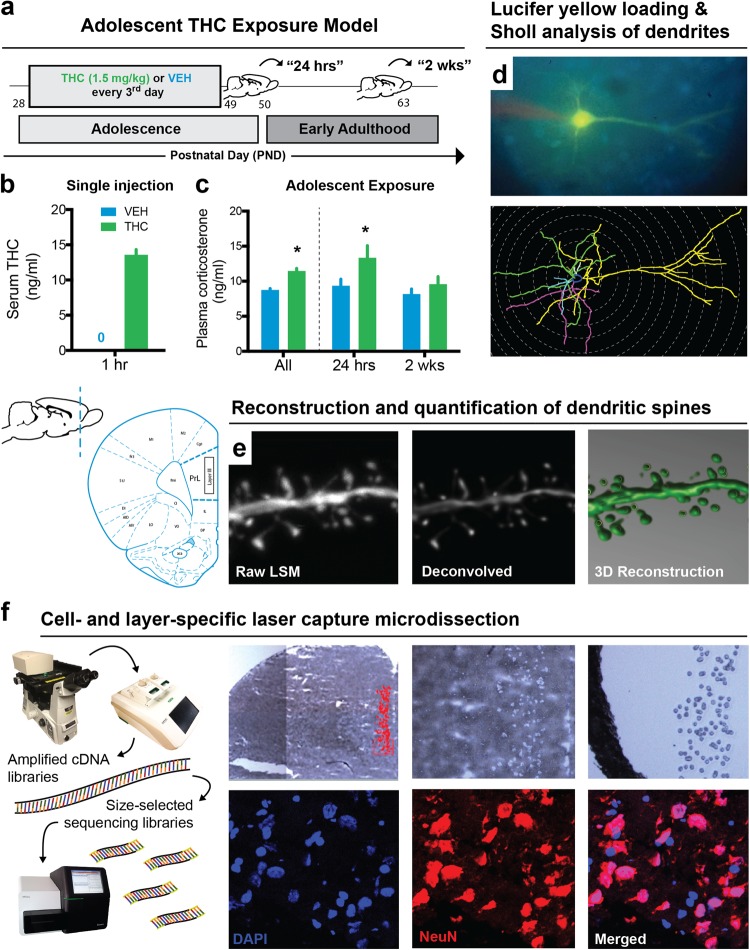
Model of adolescent THC exposure and approaches for layer- and cell-specific morphological and transcriptional profiling of prelimbic (PrL) pyramidal neurons. **a** Schematic timeline of THC exposure during adolescence and developmental time points studied. **b** Acute administration of 1.5 mg/kg THC increased circulating serum THC concentration 1 h after injection. **c** Adolescent exposure to THC elevated corticosterone levels, most notably 24 h after the last administration that may reflect a component of acute withdrawal. **d**–**f** Multidisciplinary approach to study the developmental effects of THC on pyramidal neuron morphology and gene expression in layer III of the rodent PrL. Dendritic branching **d** and spine density **e** were studied by filling pyramidal neurons with Lucifer yellow. (**f**) Pyramidal neurons from layer III were microdissected from Nissl-stained sections and sequenced for downstream transcriptome-wide profiling

Beginning on postnatal day (PND) 28, male Long-Evans rats were randomly assigned to receive intraperitoneal injections of either THC (1.5 mg/kg) or control (vehicle (VEH) containing 0.9% NaCl in sterile water with 0.3% Tween 80) solution every third day for a total of eight injections (Fig. 1a) [32]. This THC dose acutely raised serum concentrations to levels comparable to acute cannabis intoxication in humans [33] (Fig. 1b) and intermittent exposure of this dose during adolescence maintained elevated plasma corticosterone (Fig. 1c). Brains and blood were collected 24 h (PND 50) or 2 weeks (PND 63) after the last injection.

### Microscopic neuromorphology

Cells loaded with 5% Lucifer yellow were imaged to assess branching complexity (Fig. 1d) and spine density (Fig. 1e) [34–36]. Dendritic trees were reconstructed under fluorescent illumination (× 40) using Neurolucida software (MBF Biosciences) and branching complexity was quantified using the Sholl analysis (*n* = 6–9 rats/group, five cells per rat) [37]. Dendritic branches were sampled on a confocal microscope (× 100) with ZEN software (Zeiss) and reconstructed with NeuroStudio [38].

### LCM and RNA-sequencing

Cells from Nissl-stained sections were captured using an ArcturusXT LCM microscope (ThermoFisher). Although cells from layer II–III cells were sampled, as most studies often do not distinguish these layers, the captured cells were predominantly taken from layer III. After collecting lysate from all samples (*n* = 4–5 rats/group), RNA was extracted with TRIzol, then reverse-transcribed into complementary DNA (cDNA) and amplified using the REPLI-g Whole Transcriptome Amplification Single-Cell Kit (Qiagen). Sequencing libraries, bar-coded for the Illumina MiSeq and HiSeq platforms, were generated from 1 µg sonicated and purified cDNA, then sequenced-by-synthesis using 50-bp single-end chemistry.

### Transcriptome analysis

A standard RNA-sequencing (RNA-Seq) analytic pipeline was used for quality control, alignment, and gene counting (https://github.com/shenlab-sinai/SPEctRA). Differential gene expression between pyramidal and non-pyramidal cells, and in the context of normal development (24 h vs. 2 weeks in VEH-treated animals), developmental THC (24 h vs. 2 weeks in THC-treated animals), and simple treatment main-effects at each time point, were calculated using the Bioconductor package voom-limma [39] in R. Subsequently, weighted gene co-expression network analysis (WGCNA) was performed on all samples combined to identify co-expression modules (arbitrarily labeled by colors such as dark-grey, green-yellow, turquoise, etc.). Each module represents a group of genes whose expression patterns are similar across the samples and presumably coregulated. The statistical significance of overlap between two gene modules/lists was assessed in *R* using the Bioconductor GeneOverlap package [40].

### CommonMind Consortium (CMC) analysis

RNA-seq data was obtained from the dorsolateral PFC of 258 schizophrenia and 279 control subjects [41]. The gene membership of control and schizophrenia CMC modules was predetermined based on previously published data and used to test enrichment with developmental THC modules.

### Statistical analysis

For morphological data, either between-subjects 2 × 2 ANOVAs, or mixed-design 2 × 2 × *n* ANOVAs, were used to compare treatment (VEH and THC) and time (24 h and 2 weeks), whereby *n* was the within-subjects factor representing distance from soma (known as the Sholl interval) and contained either four (basal) or eight (apical) levels.

## Results

### Adolescent THC exposure altered the developmental trajectory of dendritic arbors

**Fig. 2:**
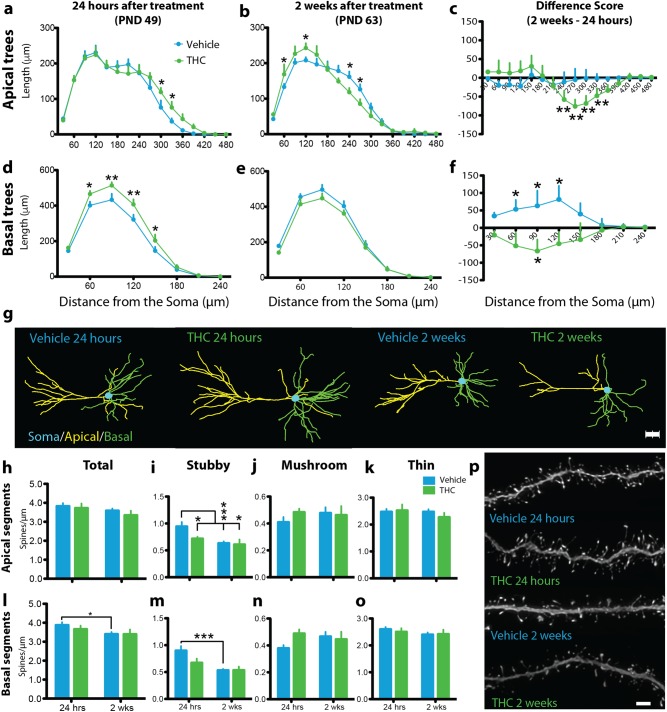
Adolescent THC exposure altered the dendritic arborization, spine density, and developmental trajectory of layer III prelimbic (PrL) pyramidal neurons. **a** Adolescent THC significantly increased distal apical dendritic arborization in early adolescence (24 h after drug treatment). **b** In early adulthood (2 weeks after drug treatment), a significant allostatic reversal resulting in atrophy was observed in distal apical trees accompanied by the emergence of significantly increased proximal branching. **c** Between time points, vehicle-treated animals (VEH) exhibited no difference in apical arbor complexity while THC-treated animals (THC) exhibited a significant decrease in distal apical branching complexity. **d** Adolescent THC exposure significantly increased basal dendritic arborization 24 h after drug treatment. **e** Two weeks after drug treatment, a non-significant allostatic reversal was observed in basal trees. **f** Vehicle-treated animals exhibited significantly increased basal arbor complexity between time points, whereas THC-treated animals exhibited significantly decreased basal apical branching complexity. **g** Representative dendrograms of traced PrL pyramidal neurons. **h** No significant differences were observed in total apical dendritic spine density. **i** Vehicle-treated subjects exhibited significant pruning of stubby apical dendritic spines between time points, whereas THC-treated subjects exhibited premature pruning. **j** No significant differences in apical mushroom spine density were observed. **k** No significant differences in apical thin spine density were observed. **l** Vehicle-treated subjects exhibited significant pruning of total basal dendritic spines between time points, whereas THC-treated subjects exhibited premature pruning. **m** Vehicle-treated subjects exhibited significant pruning of stubby basal dendritic spines between time points, whereas THC-treated subjects exhibited premature pruning. **n** No significant differences in basal mushroom spine density were observed. **o** No significant differences in basal thin spine density were observed. **p** Representative deconvolved basal dendritic branches from all treatment conditions. *P ≤ 0.05, **P ≤ 0.01, ***P ≤ 0.001. Data shown as mean + SEM **a**–**b**, **d**–**e**, **h**–**o**. Data represent the difference between 2 weeks and 24 h, shown as mean + standard error of the difference between treatments **c**, **f**. Scale bar = 2 μm **p**

To investigate the morphological consequences of adolescent THC exposure, we reconstructed the apical and basal dendritic trees of layer III PrL pyramidal neurons (Fig. 1d). Arbor development was markedly reorganized by drug exposure. There was no difference in total dendritic length in apical or basal trees at either time point (Supplementary Fig. 1), but Sholl analysis detected interactions in apical (treatment×time×Sholl interval interaction: *F*_15,420_ = 2.1, *P* = 0.009, Cohen’s *d* = 0.76–1.19 for significant pairwise comparisons) and basal (treatment×time×Sholl interval interaction: *F*_7,196_ = 2.71, *P* = 0.01, Cohen’s *d* = 0.98–1.15 for significant pairwise comparisons) trees indicating that development and THC exposure altered dendritic arbor complexity (Fig. 2a–g, Supplementary Video 1). Across development, VEH-treated subjects exhibited stable apical trees (Fig. 2c) but expanded complexity of basal trees into adulthood (Fig. 2f). This pattern differed significantly for adult rats with a history of adolescent THC exposure insomuch that this treatment increased arborization in distal apical and basal trees 24 h after the last injection (Fig. 2a, d). In early adulthood, however, 2 weeks after drug exposure, THC-treated animals showed significant atrophy in distal apical arbors beginning at 270 μm from the soma and increased proximal apical arborization between 60 μm and 120 μm from the soma (Fig. 2b). Moreover, only THC-treated rats exhibited a reduction in basal tree complexity from adolescence to adulthood (Fig. 2f) emphasizing the marked reorganization of arbor development induced by drug exposure.

### Adolescent THC exposure prematurely prunes dendritic spines

In addition to the complexity of dendritic trees, the spines on arbor branches are a central morphological feature of synaptic plasticity involved in the maturation of the cortex and implicated in the pathophysiology of neuropsychiatric disorders [42, 43]. We investigated the consequences of adolescent THC exposure on dendritic spines by reconstructing branches of dendritic arbors and quantifying the type—mushroom, stubby, or thin—and density of dendritic spines (Fig. 1e). Only stubby spines showed significant developmental fluctuation and sensitivity to THC exposure. VEH-treated animals exhibited reduction in apical stubby spine density (Fig. 2h–k) between adolescence and adulthood (*t*_15_ = 3.90, *P* = 0.001, Cohen’s *d* = 1.78) consistent with pruning normally seen during this developmental period. This pattern was absent in THC-treated rats (*t*_9_ = 0.27, *P* = 0.268, Cohen’s *d* = 0.72). Instead, reduced stubby spine density was already evident in THC-treated rats compared with VEH-treated rats 24 h after the last injection (*t*_11_ = 2.35, *P* = 0.039, Cohen’s *d* = 1.31) and those levels remained low into adulthood. A similar pattern was observed in basal arbors (Fig. 2h–p), which showed a marked reduction in stubby spine density between time points in VEH-treated (*t*_15_ = 4.82, *P* < 0.001, Cohen’s *d* = 2.37), but not THC-treated (*t*_9_ = 1.29, *P* = 0.228, Cohen’s *d* = 0.78), animals. These findings suggest a morphological correlate of adolescent THC-mediated alterations of PFC structure observed in humans [14–16].

### Analysis of PrL pyramidal neuron and non-pyramidal cell transcriptome

**Fig. 3:**
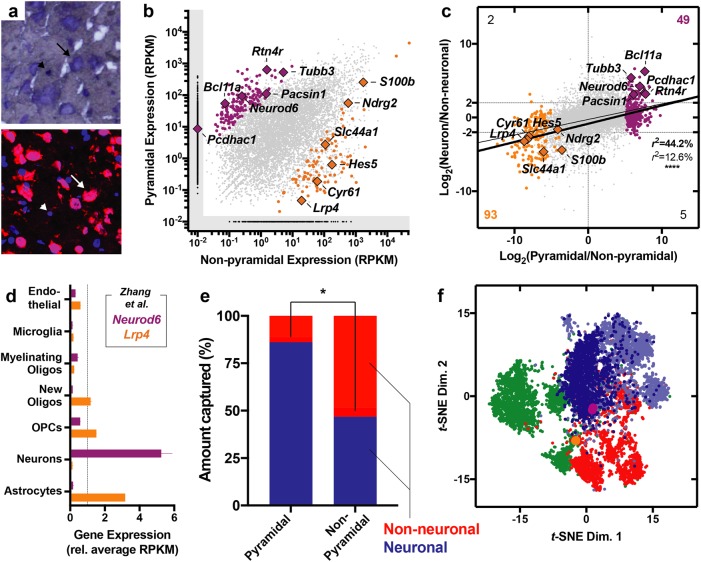
Laser capture microdissection (LCM) and RNA-sequencing of pyramidal neurons and non-pyramidal cells. **a** Representative micrographs highlighting Nissl-stained frozen tissue (top) and NeuN immunohistochemistry (bottom). Arrows indicate pyramidal neurons and arrowheads indicate adjacent non-pyramidal, NeuN- cells. **b** Scatter plot displaying genes expressed and statistically enriched in pyramidal (purple) and non-pyramidal (orange) populations (*P* < 0.01). **c** Scatter plot displaying relationship between pyramidal enrichment pattern (*x* axis, LCM) and neuronal enrichment (*y* axis, sorted mouse neocortex [44]). Thin line indicates regression with all genes and thick line indicates regression of with only differentially expressed (colored) genes. Counts in each corner indicate number of genes within the respective quadrant that were both statistically enriched (*P* < 0.01 based on LCM) and showed at least a 2^2^-fold enrichment pattern (based on sorting). **d** Data from Zhang et al. [44]. highlighting the enrichment pattern of *Neurod6* and *Lrp4*, enriched in neuronal and non-neuronal cells, respectively. **e** In silico cytometry revealed enrichment of neuronal cells within the pyramidal population and a relative depletion of this cell type in the non-pyramidal population (*P* < 0.05). **f** Non-linear cluster analysis highlighted the topological relatedness between the pyramidal population (purple) and excitatory cortical neurons (blue) from the human neocortex, especially the Ex1 subtype (dark blue), which reflects layers II and III cortical projection neurons as defined by Lake et al. [45]. The non-pyramidal cells (orange), on the other hand, were more similar to glia (green) and inhibitory cortical neurons (red)

Based on the morphological impairments induced by THC, we next wanted to expand insights about the molecular make-up of the PrL cells. As such, we developed a strategy to integrate layer-specific LCM and next-generation RNA-seq to measure the transcriptome of morphologically distinct layer III cellular populations (Fig. 1f). We confirmed cell type specificity by capturing and sequencing morphologically identified pyramidal and non-pyramidal populations (Fig. 3a). Sequencing revealed 9740 genes that met criteria for detection. Between these distinctively captured cellular populations, 327 (3.4%) genes were significantly enriched in pyramidal population, whereas only 187 genes (1.9%) were enriched in the non-pyramidal populations, presumably reflecting the latter fraction’s more heterogeneous composition (Fig. 3b, Supplementary Table 1). When holistically comparing the enrichment pattern of the LCM-derived pyramidal population to sorted mouse neocortical neurons (Fig. 3c, Supplementary Table 1), there was a statistically significant linear regression between the fold differences (*r*^2^_8236_ = 0.126, *P* < 0.0001) that was especially pronounced when only differentially expressed genes are included in the analysis (*r*^2^_461_ = 0.442, *P* < 0.0001). Among these, consistently differentially expressed genes included *Neurod6*, which was enriched in the pyramidal population, and *Lrp4*, which was enriched in the non-pyramidal population (Fig. 3d) [44]. In silico cytometry, performed using CIBERSORT, confirmed that the pyramidal population was significantly more neuronal than the non-pyramidal population (*t*_2_ = 6.67, *P* = 0.022), the latter being an admixture of neuronal and glial components (Fig. 3e). Furthermore, non-linear cluster analysis using *t*-SNE highlighted the topographical relatedness between the pyramidal population and human excitatory neocortical neurons (Fig. 3f), especially cortical projection neurons from layers II and III (Ex1 subtype) defined by Lake et al. [45]. On the other hand, this analysis highlighted that the non-pyramidal population was more related to inhibitory cortical neurons and non-neuronal cortical cells (Fig. 3f). Overall, these results validated the technical approach by highlighting this strategy’s ability to characterize cell- and layer-specific transcriptional landscapes.

### Short-term and protracted effects of adolescent THC on the transcriptome of PrL pyramidal neurons

After confirming our capacity to selectively isolate and sequence morphologically defined PrL cellular populations, we interrogated the transcriptomic profile of layer III PrL pyramidal neurons in the rat adolescent THC model. Sequencing revealed 12,568 genes that met criteria for detection and in silico cytometry for each animal revealed that the microdissected material derived predominantly from neurons (93.5 ± 0.6%), suggesting minimal contamination from non-neuronal sources (6.5 ± 0.2%) with no difference between the groups (all *P* > 0.05; Supplementary Fig. 2).

Evaluation of the adolescent and young adult time points identified genes associated with the early and protracted effects of THC, respectively. As compared with VEH-treated animals, 698 (5.6%) differentially expressed genes were identified in the THC-treated animals 24 h after their last injection (Supplementary Fig. 3a, Supplementary Table 2). Gene enrichment analysis revealed that these genes related to cellular response to organonitrogen compound and Cul3-RING ubiquitin ligase complex (Supplementary Fig. 3a). In early adulthood, 2 weeks after the animals’ last injection, 608 (4.8%) differentially expressed genes were identified in THC-treated animals compared with controls (Supplementary Fig. 3b, Supplementary Table 2). In contrast to the early changes, THC had protracted effects on gene networks associated with microtubule organization and cytochrome complex assembly (Supplementary Fig. 3b).

### Adolescent THC alters the normal developmental trajectory of the PrL pyramidal transcriptome

**Fig. 4:**
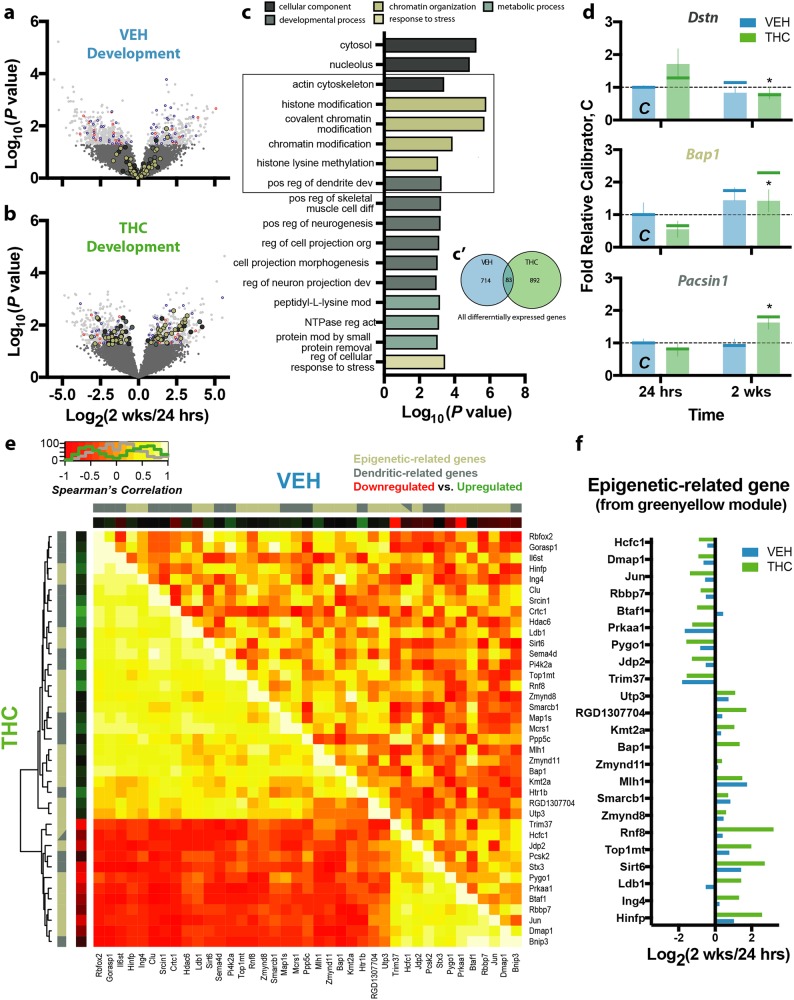
Effect of normal development and THC exposure on transcriptional landscape. **a**–**b** Development in vehicle-treated animals **a** resulted in 797 differentially expressed genes, whereas development in THC-treated animals **b** resulted in 975. Genes differentially expressed in both contrasts are colored blue (similar direction) or red (opposite direction), whereas other colors correspond to the biological categories shown in **c**. **c** Genes differentially expressed by developmental THC, which minimally overlapped with those differentially expressed in VEH-treated animals **c’**, engaged biological processes related to development, chromatin organization, and metabolism, as well as pathways related to cytosolic and nucleolar components. **d** Representative genes from these pathways included *Dstn* (top, actin cytoskeleton), *Bap1* (middle, chromatin organization), and *Pacsin1* (bottom, developmental processes), whose differential expression were replicated using NanoString (indicated by vertical bars with RNA-seq data shown as vertical lines for comparison). **e** Epigenetic- and dendritic-related genes within the WGCNA green-yellow module were significantly co-expressed in the THC-treated animals (THC) insomuch that a subset of genes were positively correlated, whereas a majority were anticorrelated. This pattern of coordinated expression was, however, not observed in the vehicle-treated animals (VEH). **e** Differential expression of the epigenetic-related genes from the WGCNA green-yellow module. *P ≤ 0.05, relative THC at 24 h. Data shown as mean ± SEM

Based on the altered trajectory of morphological complexity seen after adolescent exposure to THC, a key question was whether THC exposure affected the transcriptional ontogeny of PrL neurons. Across development, from adolescence to early adulthood, 797 (6.3%) genes were significantly different in the adult VEH-treated animals when compared with the younger control animals (Fig. 4a, Supplementary Table 2). Instead, 975 (7.8%) genes were significantly different in the adult THC-treated animals (Fig. 4b, Supplementary Table 2) when compared with the younger THC-treated animals. Relative to the biological processes engaged by normal development, genes dysregulated by developmental THC uniquely disrupted epigenetic mechanisms (Fig. 4c). Specifically, VEH-treated animals exhibited changes in genes associated with signal transduction, cytoskeletal protein actin projection protrusion (lamellipodium), and cell morphogenesis, all as expected during normal development (Supplementary Fig. 4), whereas THC-treated animals exhibited changes in genes associated with, not only actin cytoskeleton and dendritic regulation, but also chromatin modification and histone methylation (Fig. 4c). Surprisingly, there was little overlap in these developmentally dynamic genes, with only 83 (4.9%) shared between the VEH- and THC-treated developmental contrasts (Fig. 4c). Within the pathways dysregulated by developmental THC, top differentially expressed genes, replicated using NanoString (Supplementary Fig. 5, Supplementary Table 3), included *Dstn*, *Pacsin1*—which was highly enriched in the pyramidal fraction (log_2_FC = 6.17, *P* = 0.006, Supplementary Table 1)—and *Bap1* (Fig. 4d). These data demonstrate that cannabinoids profoundly disrupt the developmental trajectory of layer III PrL pyramidal neuron’s transcriptome.

Marked disturbances associated with histone modification and chromatin remodeling were observed across development in THC-treated animals (Supplementary Table 4). Enrichment analyses of the differentially expressed genes indicated the strongest functional association with *Kmt2a* (also known as *Mll1*) and histone H3 lysine 4 trimethylation (H3K4me3). Interestingly, *Kmt2a*—a chromatin methyltransferase—mediates its activity specifically at H3K4 and is highly implicated in cellular processes linked to neurodevelopment and psychiatric disorders [46].

To assess the developmental effects of THC on coordinated transcriptional expression, WGCNA on the RNA-seq data was performed. Developmental THC was significantly associated with one module (dark-grey, *P* = 0.012), whereas four other modules were developmentally regulated (green-yellow, *P* = 8.5 × 10^−4^; tan, *P* = 7.79 × 10^−3^; green, *P* = 0.0082; skyblue, *P* = 0.027). Pathway analysis (MetaCore; Thomson Reuters) of the most significant developmentally regulated module—green-yellow—revealed significant enrichment of genes related to organelle organization (103 genes, *P* = 1.18 × 10^−11^), cellular component organization (146 genes, *P* = 2.38 × 10^−11^), chromatin modification (33 genes, *P* = 9.84 × 10^−11^) and histone modification (24 genes, *P* = 2.63 × 10^−9^; Supplementary Table 5). Interestingly, several genes within this module were significantly affected by THC across development including the histone acetyltransferase *Jade2* (log_2_FC = 5.65, *P* = 2.2 × 10^−5^), histone arginine methyltransferase *Prmt5* (log_2_FC = 1.66, *P* = 0.013)—which was highly enriched in the pyramidal fraction (log_2_FC = 6.82, *P* = 0.001, Supplementary Table 1)—and transcription factor *Cebpg* (log_2_FC = −1.43, *P* = 0.002).

We further investigated the co-expression between epigenetic modifiers and dendritic regulators within the green-yellow module (23 and 17 genes, respectively) to identify THC-dependent interactions between these two relevant categories. Using non-parametric Spearman’s rank correlation, 30.02% of all possible gene-gene pairs showed significant correlations (at *P* < 0.05) in the THC-treated animals compared with only 5.95% in the controls (Fig. 4e), suggesting a strong functional relationship between epigenetic mechanisms and dendritic morphology specific to developmental THC exposure. Indeed, substantially more chromatin modifiers from this module were developmentally regulated in THC-treated animals (7/23 genes) than in controls (1/23 genes), again indicating a strong THC-related impairment of epigenetic regulation during development (Fig. 4f).

### Developmentally regulated THC gene networks overlap those in schizophrenia subjects

**Fig. 5:**
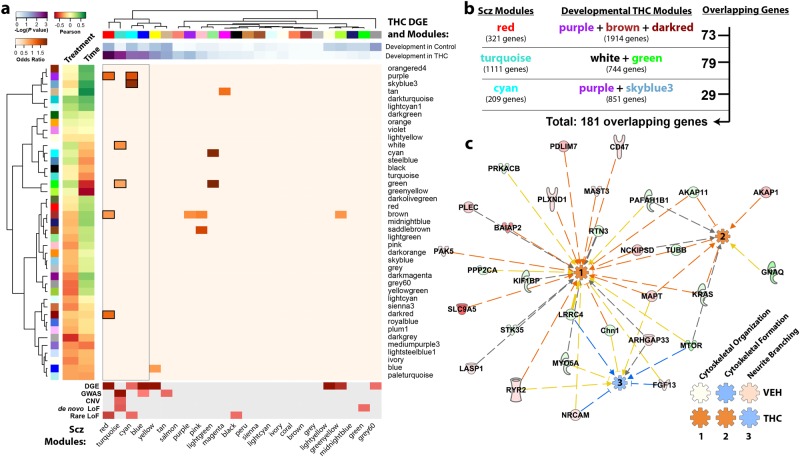
Genes developmentally dysregulated by adolescent exposure to THC are also dysregulated in the DLPFC of humans with schizophrenia. **a** Enrichment analysis shows significant overlap between genes differentially expressed by developmental THC and co-expression modules identified in human schizophrenia. In addition, these schizophrenia CommonMind Consortium (CMC) modules overlapped with developmental THC co-expression modules further suggesting a common epigenetic landscape. **b** Pathway analysis was performed on an overlapping set of 181 genes to further explore the biological relevance of these shared genes. **c** Ingenuity pathway analysis revealed that these genes are predicted to enhance cytoskeletal function (organization and formation) and suppress neurite branching in the THC-treated animals, but not in the vehicle-treated animals (see Supplementary Fig. 7), based on differential expression

Given the relationship between cannabis use, PFC development, and schizophrenia, we evaluated the relationship between genes dysregulated by developmental THC and human schizophrenia. We interrogated the CMC sample data set consisting of 537 subjects (258 subjects with schizophrenia and 279 controls) with dorsolateral PFC RNA-seq data [41]. Comparing developmentally dysregulated genes (based on differential gene expression analysis) to genes coregulated in schizophrenia (termed schizophrenia CMC modules), genes developmentally dysregulated by THC overlapped significantly with the red (OR = 1.92, *P* = 9.50 × 10^−4^), turquoise (OR = 1.42, *P* = 3.05 × 10^−3^), cyan (OR = 1.82, *P* = 0.012), blue (OR = 1.47, *P* = 0.012), yellow (OR = 1.43, *P* = 0.028), and tan (OR = 1.57, *P* = 0.048) schizophrenia CMC modules (Fig. 5a, upper heatmap, Supplementary Table 6). The top three schizophrenia CMC modules—red, turquoise, and cyan—were especially noteworthy as they each significantly overlapped with multiple developmental THC co-expression modules (identified in WGCNA), further suggesting that developmental THC dysregulates PFC expression of schizophrenia-associated genes (Fig. 5a, center heatmap, Supplementary Table 6). In support of this overlap analysis, these three schizophrenia modules were significantly enriched for genes involved in retrograde eCB signaling, glutamatergic signaling, as well as axonal guidance, synaptic transmission, and neuroplasticity (Supplementary Fig. 6). Interestingly, the red, turquoise, cyan, and blue schizophrenia CMC modules were the most dysregulated networks among cases with schizophrenia (Fig. 5a, lower heatmap) [41]. These data highlight similarities in network perturbations owing to early THC exposure and schizophrenia.

To identify shared sets of co-expressed genes in an agnostic fashion, our subsequent pathway analysis focused on the genes common to the overlapping schizophrenia and developmental THC modules (at *P* < 0.01). From the 181 genes that met criteria (Fig. 5b), there was a significant enrichment for cytoskeletal and neurite development, which were predicted to be activated and deactivated, respectively (Fig. 5c). The predicted deactivation in neurite development is particularly noteworthy given that animals exposed to developmental THC exhibit decreased dendritic branching. In control animals, on the other hand, neurite branching was activated (Fig. 5c inset, Supplementary Fig. 7) consistent with the increased dendritic branching observed in normal animals during the developmental window studied.

## Discussion

Prefrontal pyramidal neurons comprise circuits that modulate mesolimbic function dysregulated in psychiatric disorders. Developing a novel strategy that enabled cell type- and layer-specific transcriptomic analyses, we obtained unique insights on the structural and molecular phenotype of layer III PrL pyramidal neurons in this critical node of mesolimbic and cortical function. We report that adolescent THC exposure induces distinct proximate and long-term alterations of dendritic architecture, reducing neuronal complexity and reprogramming the transcriptional landscape of layer III PrL pyramidal neurons. Transcriptional abnormalities of synaptic plasticity and neuropathological alterations in pyramidal neuron dendritic arbors and spines are widely implicated in psychiatric disorders such as schizophrenia [43] and are likely relevant to the association between adolescent cannabis use and psychiatric vulnerability [47].

The dynamic nature of adolescent PFC development renders this brain region sensitive to external and internal factors that may impact mental health later in life. VEH-treated animals in our study exhibited stable apical tree architecture, expansions in basal tree arborization, and pruning of dendritic spines as would be expected during typical development [48]. On the other hand, THC exposure disrupted this developmental pattern with premature pruning of spines and protracted atrophy of distal apical trees. Interestingly, chronic stress induces similar atrophy of distal PrL pyramidal neuron apical trees [35, 49], and enhanced stress and anxiety are characteristic symptoms reported by humans using and withdrawing from cannabis [50]. Synthetic cannabinoid exposure during adolescence was also recently shown to induce distinct changes in the dendritic morphology, for instance with CP55,940 primarily reducing the basal branches’ complexity [51]. Intriguingly, the effects of THC we observed on spines were fundamentally different from arbors, as the latter exhibited an allostatic reversal into adulthood, whereas THC prematurely decreased spine density with no compensatory alterations later in life.

Pruning of the PFC during adolescent development is a conserved phenomenon seen across species including rodents, monkeys, and humans [52]. Our findings indicate that loss of stubby spines was the predominant driver of pruning during normal adolescent development. Stubby spines, like thin spines and shaft synapses, have greater actin motility than mushroom spines, thus contributing to enhanced plasticity during development [53]. The persistent reduction of stubby spines by adolescent THC exposure prematurely attenuates the capacity for plasticity in circuits refining themselves for adulthood. Interestingly, reduction of stubby spines is detected in the mPFC of individuals with post-traumatic stress disorder [54] and in hippocampus of animals modeling adolescent social defeat stress [55]. In female rats exposed to THC during adolescence, early reductions in spine density are also observed, but unlike in our study, these reductions persist into adulthood, which may be owing to differences in study design such as gender or dosing schedules [56]. It is noteworthy to recognize that these changes in morphology may not be unique to adolescent exposure, as male rats exposed during adulthood exhibit changes in layer V pyramidal neuron morphology [57]. Nonetheless, although the morphological phenotypes and underlying mechanisms shared by different exposure periods remain unknown, cannabis use remains exceedingly prevalent in adolescents, which further emphasizes the needed focus on this vulnerable (at-risk) patient population.

The LCM RNA-seq strategy we developed to profile the discrete molecular phenotype of layer III cells revealed that pyramidal neurons in THC-treated animals were characterized by marked reprogramming of the transcriptome, concomitant with morphological disturbances. Only ~ 5% of all differentially expressed genes overlapped between VEH- and THC-treated animals suggesting that THC vastly perturbed the developmental trajectory of PrL transcriptional maturation. Specifically, the gene networks altered by adolescent THC exposure involved regulation of actin dynamics at excitatory synapses and dendritic spines. Several genes showing enhanced THC sensitivity across development, notably pyramidal-enriched *Pacsin1* [58], as well as *Clu* [59] and *Snap25* [60], are also implicated in psychiatric diseases such as schizophrenia and mood disorders. Intriguingly, the coordinated expression of genes altered by THC across development overlapped with modules dysregulated in the PFC of humans with schizophrenia and may reflect the morphological disturbances caused by THC exposure. For example, functional networks related to branching of neurites that were activated in control animals across development were deactivated by adolescent THC exposure, recapitulating the morphological observations of reduced dendritic branching seen in these groups. It is possible that the overlapping networks are secondary to changes in gene expression owing to previous marijuana use by the schizophrenia subjects.

In addition to the dysregulation of genes central to cytoskeletal organization and synaptic function, the most developmentally dynamic networks were histone and chromatin modifications. Accumulating evidence in human and animal models has begun to elucidate epigenetic contributions to the developmental effects of cannabis [61]. Co-expression analyses in the current study underscored a strong relationship between genes related to epigenetic modification and those linked to synaptic plasticity in THC-exposed animals. The strongest functional association highlighted Kmt2a and H3K4me3 as prominent modifications. This finding is intriguing given that MLL1, an H3K4 methyltransferase, is essential for neurogenesis [62] and PFC synaptic plasticity [63], is a key regulator of morphogenesis [64] and postmortem human studies and animal models have suggested its involvement in schizophrenia-related cortical dysfunction [65]. Future studies are needed to establish the causal effects of these epigenetics factors, and their downstream targets, on pyramidal neuron morphology and behaviors affected by THC. Moreover, characterizing molecular alterations within other cortical cells will provide important insights regarding possible discrete signatures within specific cell types associated with the long-term impact of adolescent THC exposure.

In summary, the current study provides direct evidence that exposure to THC during adolescence alters the trajectory of developing mPFC circuitry. Adolescent THC exposure, by reshaping PFC pyramidal neurons on a molecular and morphological level, may alter psychiatric vulnerability particularly in individuals with overlapping genetic disturbances within THC-sensitive gene networks linked to cytoskeletal architecture and synaptic plasticity.

## Electronic supplementary material

Supplemental Materials and Methods

Supplementary Video 1

Supplementary Table 1

Supplementary Table 2

Supplementary Table 3

Supplementary Table 6
