# Supplementary material for: Adolescent exposure to Δ9-tetrahydrocannabinol alters the transcriptional trajectory and dendritic architecture of prefrontal pyramidal neurons
Source: Mol Psychiatry. 2018 Oct 3;24(4):588–600. doi: 10.1038/s41380-018-0243-x (PMC6430678; doi:10.1038/s41380-018-0243-x)
Supplement: Supplementary file 1 — Supplemental Materials and Methods [file 41380_2018_243_MOESM1_ESM.docx]

**Adolescent exposure to Δ^9^-tetrahydrocannabinol alters the transcriptional trajectory and dendritic architecture of prefrontal pyramidal neurons**

Michael L. Miller,^1,2,3,4,6^ Benjamin Chadwick,^1,2,3,4,6^ Dara L. Dickstein,^1,4^ Immanuel Purushothaman,^1,4^ Gabor Egervari,^1,2,3,4^ Tanni Rahman,^1,2,3,4^ Chloe Tessereau,^1,2,4^ Patrick R. Hof,^1,4^ Panos Roussos,^2,4,5^ Li Shen,^1,4^ Mark G. Baxter,^1,4^ and Yasmin L. Hurd^1,2,4^

^1^Fishberg Department of Neuroscience, ^2^Department of Psychiatry, ^3^Graduate School of Biological Sciences, ^4^Addiction Institute at Mount Sinai, Friedman Brain Institute and ^5^Institute for Genomics and Multiscale Biology, Department of Genetics and Genomic Sciences — all at the Icahn School of Medicine at Mount Sinai, New York, NY, USA

^6^These authors contributed equally to this work.

# MATERIALS AND METHODS

## Tissue collection

Male Long-Evans rats (Charles River Laboratories; Raleigh, NC, USA) were received at postnatal day (PND) 21 and pair housed in a temperature-controlled environment with *ad libitum* access to food and water. All procedures were approved by the Bronx Veterans Affairs Hospital and Icahn School of Medicine at Mount Sinai Institutional Animal Care and Use Committee. Animals were treated with either THC or a saline-based vehicle solution. THC preparation and treatment was performed utilizing our standardized paradigm of eight intraperitoneal injections of 1.5 mg/kg THC (National Institute on Drug Abuse Drug Supply Program, Bethesda, MD, USA) or vehicle solution every third day beginning on PND 28 (Fig. 1a).^1^ This treatment produces circulating serum THC concentration comparable to acute intoxication in humans^2^ and mimics the sporadic use typical of adolescent THC consumption (Fig. 1b). Sample size chosen based on experience with methodology and in order to achieve adequate number of neurons suitable for morphological analysis.

Brains were collected 24 hours (PND 50) or 2 weeks (PND 63) after the last THC injection (Fig. 1a). Fixed tissue for cell loading was collected following intracardial perfusion with 4% paraformaldehyde (PFA), sliced into 250 μm-thick coronal sections with a VT1000S Vibratome (Leica; Buffalo Grove, IL, USA) and stored at 4°C in 10 mM phosphate-buffered saline (PBS) containing 0.1% sodium azide. Dissected whole brains for LCM and immunohistochemistry were immediately submerged in isopentane, cooled on dry ice, then stored at -80°C until cryosectioning. Three consecutive series of 20 coronal sections (10 μM thick) throughout the entire frontal cortex were collected with a Frigocut 2800E cryostat (Leica Instruments; Nobloch, Germany) onto uncharged glass slides and stored at -30°C.

## ELISA/EIA

For THC ELISA, trunk blood was collected 1 hour after a single dose of THC (1.5 mg/kg) or vehicle in THC-naïve PND 50 animals. Phase-separated serum was tested for the presence of THC using the MaxSignal® THC ELISA kit according to the manufacturer’s instruction (Bioo Scientific Corporation; Austin, TX, USA). For corticosterone EIA, trunk blood was directly collected into heparinized, green-topped vacutainers 24 hours or 2 weeks after adolescent exposure to THC (1.5 mg/kg) or vehicle. Blood samples were gently mixed and briefly kept on ice until they were transferred to microcentrifuge tubes and centrifuged at room temperature for 15 minutes at 2000 rcf. The resulting phase-separated, platelet-depleted plasma was collected and plasma corticosterone was measured from 5 µl per sample using the Limited Corticosterone HS (High Sensitivity) EIA kit (ref no. AC-15F1) according to the manufacturer’s instruction (Immunodiagnostic Systems; NE35 9PD, United Kingdom).

## Cell loading and microscopy

Cell loading, tracing, and imaging of dendritic branches were performed on fixed brain tissue as previously described.^3-8^ A micropipette inserted into pyramidal neurons in layer III of the PrL injected 5% Lucifer yellow (Invitrogen; Carlsbad, CA, USA) in deionized water via iontophoresis at 5-10 nA (Fig. 1d). Sections were mounted in FluoroMount-G (BD Diagnostic Systems; Franklin Lakes, NJ, USA) and stored at 4°C. An Axiophot 2 microscope (Zeiss; Oberkochen, Germany) with a 40X objective (Plan-NEO, 1.3 N.A.) was used to reconstructed 5 pyramidal neurons per rat with Neurolucida software (MBF Biosciences; Williston, VT, USA). Sholl analyses^9^ to quantify branching complexity were performed with NeuronExplorer software (MBF Biosciences) on apical and basal arbors. Secondary, tertiary and quaternary dendritic branches were imaged on an inverted Zeiss 510 confocal microscope using a 100X objective (1.4 NA oil-immersion) with ZEN software (Zeiss) at voxel dimensions 0.05 × 0.05 × 0.10 µm. An average of 14 apical and 11 basal dendritic branches per rat were sampled at the resolution required for reconstruction. Experimenters blind to treatment performed all cell loading, microscopy and analysis.

## Immunohistochemistry

Experiments were performed on fresh frozen tissue. Washes and incubation were performed at room temperature unless otherwise specified. Slides were post-fixed in 4% PFA (10 mins) then washed 3 times in PBS. Slides were placed in methanol (10 mins) at -20°C, washed in PBS (3 times) and incubated in blocking solution, consisting of 10% Normal Goat Serum, PGBA buffer (PBS/0.1% gelatin/1% BSA/0.1% sodium azide) and 0.3% Triton-X, for 1 hour. Mouse anti-NeuN antibody (cat no. MAB377; Millipore; Darmstadt, Germany) was diluted 1:500 in blocking solution and incubated on slides overnight at 4°C. Slides were washed in PBS (3 times) and incubated in goat anti-mouse IgG Alexa Fluor 594 (ThermoFisher; Waltham, MA, USA) diluted 1:500 in blocking solution for 1 hour. Slides were washed in PBS (3 times) and mounted in Fluoromount-G with DAPI (Southern Biotech; Birmingham, AL, USA).

## Laser-capture microdissection (LCM)

Microdissection was performed on Nissl-stained fresh frozen tissue to facilitate cell- and layer-specific identification and isolation while maintaining RNA integrity (Fig. 1f). Slide-mounted brain sections were placed directly into ice-cold 70% ethanol and rehydrated in a series of ice-cold 95%, 75%, and 70% ethanol, incubated in room temperature Cresyl violet (0.1% m/v in water), and dehydrated in a series of ice cold 75%, 95% and 100% (twice) ethanol for one minute each. Brain sections were then delipidated in ice-cold xylenes for 2 mins, vacuum dried, and immediately microdissected. Tissue was captured onto high-specificity LCM caps using an ArcturusXT® LCM (ThermoFisher). After cells were captured to the adherent thermolabile plastic, 20 μl of TRIzol was pipetted onto the cap, incubated at 37°C for 20 mins, collected via centrifugation at 800 rcf for 2 mins, and stored at 4°C. To confirm cell type specificity, 100 non-pyramidal cells and an average of 84 pyramidal neurons (number normalized to capture area for non-pyramidal cells) were captured for sequencing. For adolescent THC exposure experiments, 750 pyramidal neurons were captured from the three serial-sections, 125 per hemisphere per slide. Experimenters were blind to all experimental conditions during tissue processing and microdissection.

## RNA isolation and transcriptome amplification and library preparation

Cellular RNA was chemically extracted from TRIzol with chloroform then purified using CENTRI•spin^-20^ columns (Princeton Separations; Adelphia, NJ, USA). Purified RNA was reverse-transcribed using oligo-*d*T primers and amplified using the REPLI-g Whole Transcriptome Amplification (WTA) Single Cell Kit (Qiagen; Hilden, Germany). Amplified cDNA libraries were diluted 1:20 with RNase-free water in microcentrifuge tubes (15 µl cDNA and 285 µl water) and sheared using a Bioruptor® ultrasonicator (Diagenode; Seraing, Belgium) at 0°C for 1 hour. Sonication was carried out in four blocks (15 mins each) of sonication iterations; each block cycled between 30 sec on and 30 sec off at high power. Sheared libraries were purified into 20 µl of RNAse Free water with QIAquick PCR purification columns (Qiagen). Shearing was confirmed by bioanalysis with an 2100 bioanalyzer (Agilent; Santa Clara, CA, USA) and cDNA library concentration was quantified using a NanoDrop Spectrophotometer (ThermoFisher). Libraries for Illumina sequencing (San Diego, CA, USA) were generated from 1 µg purified cDNA using GeneRead Library Prep Kit (Qiagen) without PCR amplification then size-selected using GeneRead Size Selection kit (Qiagen).

## Gene expression

*RNA sequencing.* Size-selected Illumina sequencing libraries were PCR-quantified using GeneRead Library Quantification kit (Qiagen), diluted to 4 nM in Tris-HCl (10 mM) with Tween 20 (0.1% v/v), then pooled (lane-wise for HiSeq) in equimolar amounts. Three sheared purified samples from the developmental exposure studies did not possess high enough concentrations for library preparation and were excluded from analysis. Libraries were sequenced using 50-bp single end chemistry on an Illumina MiSeq desktop sequencer (performed in-house), or a HiSeq 2500 sequencer (New York University Genome Technology Center, New York, NY, USA), which yielded FASTQ files for each sample.

*NanoString*. A subset of 48 differentially expressed genes and 2 housekeeping genes were quantified using the hybridization-based nCounter platform (NanoString; Seattle, WA, USA). Custom barcoded probes complimentary to the 50 targets were generated by NanoString and hybridized overnight with sonicated, amplified cDNA libraries using the ChIP-String Assay, selected based on the double-stranded nature of the amplified library, according to the manufacturer’s instructions. Samples were performed in duplicate and normalized to each sample’s geometrically averaged expression of the two housekeeping genes (*Actb* and *Gapdh*). Given the *a priori* hypothesis, one-tailed *t*-tests were used to assess for the simple main-effect of treatment at each developmental time point.

## Statistical analyses

*Morphology.* Total length of apical and basal trees was compared with ANOVAs. Sholl analyses^9^ utilized 30-µm radial increments to quantify branching complexity as a function of distance from the soma (Fig. 1d). Mixed-design ANOVAs compared treatment groups and time points across the within-subjects Sholl interval (distance from soma). For post-hoc comparisons, simple treatment and time point main-effects at each Sholl interval were calculated. Since dendritic spines subtypes possess different functional properties,^10^ we examined the total density and the density of each subtype. Confocal z-stacks were deconvolved with AutoDeblur software (Media Cybernetics; Rockville, MD, USA) and imported into NeuronStudio for semi-automated spine density and type analysis (Fig. 1e).^4, 5, 11^ Spines were designated as non-stubby when the head to neck diameter ratio was >1.1. Non-stubby spines were differentiated as thin or mushroom with spines with a head diameter >0.350 µm being classified as mushroom. Between-subjects 2 x 2 ANOVAs compared apical and basal spine densities for the total spines and each subtype. Post-hoc *t*-tests compared differences when justified. All statistical testing utilized a criterion for significance of *P*≤0.05. Statistical effect sizes (estimated by Cohen’s *d*) were determined using the effsize^12^ package in *R*, and values between 0.5 and 0.8 were considered “medium” while values greater than 0.8 were considered “large”.^13^

*Cellular transcriptomes.* FASTQ files were aligned to the Rat Genome Sequencing Consortium version 6.0 (2014) *Rattus norvegicus* genome assembly (Baylor College of Medicine; Houston, TX, USA) with TopHat2/Bowtie2 (Johns Hopkins University; Baltimore, MD, USA).^14^ Aligned BAM files were counted against the Ensembl Rnor_6.0 GTF (reference) file (Cambridge, UK) using HTSeq^15^ and assigned gene names using the Bioconductor biomaRt^16^ package in R. Differential gene expression was determined using the Bioconductor voom-limma^17^ package in R for transcripts observed in ≥75% of samples in all treatment groups, or in ≥50% for the pyramidal versus non-pyramidal analysis. Transcripts differentially expressed (*P*≤0.05) in pair-wise comparison were included in enrichment analyses using GOrilla^18^ and Enrichr.^19^ Gene ontological analysis utilized a criterion for significance of *P*<0.001. To compare the transcriptome of captured pyramidal and non-pyramidal populations to sorted mouse neocortical cells,^20^ a data matrix containing absolute expression (expressed as FPKM) per purified population was downloaded (<http://web.stanford.edu/group/barres_lab/brain_rnaseq.html>) and mouse orthologues were converted into rat equivalents using the Bioconductor biomaRt^16^ package in R (refer to NCBI’s gene expression omnibus, accession GSE52564). Linear regression was used to compare the per-gene enrichment patterns observed in the two datasets. Statistical testing of overlap between THC and schizophrenia modules and/or differentially expressed genes was performed using the Bioconductor GeneOverlap^21^ package in R, with the total number of genes set to 16,178 (equaling all the genes detected in the two datasets).

*In silico cytometry and cluster analysis*. CIBERSORT,^22^ an online tool for *in silico* cytometry, was used to determine the proportion of neuronal and non-neuronal cells in each LCM sample. Published gene expression profiles, based on a microarray study of purified primary rat neuronal and non-neuronal cells,^23^ were used as reference to deconvolute each LCM sample. To generate the reference dataset, gene expression was determined directly from raw microarray CEL files (downloaded from NCBI’s gene expression omnibus, accession GSE19380) using the Bioconductor Affy^24^ package and probeset IDs were converted to rat Ensembl gene IDs using the Bioconductor Annotate^25^ package, all performed in R. The unfiltered gene list from the LCM experiment was used since the absence of a gene’s expression is important in this analysis.

To compare the transcriptomes of captured pyramidal and non-pyramidal populations to human neocortical cells, single-cell human transcriptomic data representing 10,319 cells were analyzed using the non-linear *t*-SNE algorithm. Gene expression matrix (expressed as absolute UMI counts per cell) was downloaded from NCBI’s gene expression omnibus (accession GSE71585) and human orthologues were converted into rat equivalents using the Bioconductor biomaRt^16^ package in R. Cluster analysis was restricted to the top 1000 most variable genes based on the human dataset, which was then merged with the captured pyramidal and non-pyramidal populations (two samples per population, four total), and *t*-SNE dimensions (n=4) were calculated using the Rtsne^26^ package in R.

*CommonMind Consortium (CMC) analysis.* CMC generated genotyping and RNA-seq data in the dorsolateral PFC of 258 cases with schizophrenia and 279 controls.^27^ Gene coexpression analysis was done separately for cases with schizophrenia and controls. We used published data describing the module membership of control and schizophrenia modules to test the enrichment with THC modules.

# REFERENCES

1. Dinieri JA, Hurd YL. Rat models of prenatal and adolescent cannabis exposure. *Methods Mol Biol* 2012; **829:** 231-242.

2. Grotenhermen F. Pharmacokinetics and pharmacodynamics of cannabinoids. *Clin Pharmacokinet* 2003; **42**(4)**:** 327-360.

3. Bloss EB, Janssen WG, McEwen BS, Morrison JH. Interactive effects of stress and aging on structural plasticity in the prefrontal cortex. *J Neurosci* 2010; **30**(19)**:** 6726-6731.

4. Dickstein DL, Brautigam H, Stockton SD, Jr., Schmeidler J, Hof PR. Changes in dendritic complexity and spine morphology in transgenic mice expressing human wild-type tau. *Brain Struct Funct* 2010; **214**(2-3)**:** 161-179.

5. Bloss EB, Janssen WG, Ohm DT, Yuk FJ, Wadsworth S, Saardi KM *et al.* Evidence for reduced experience-dependent dendritic spine plasticity in the aging prefrontal cortex. *J Neurosci* 2011; **31**(21)**:** 7831-7839.

6. Luebke JI, Medalla M, Amatrudo JM, Weaver CM, Crimins JL, Hunt B *et al.* Age-related changes to layer 3 pyramidal cells in the rhesus monkey visual cortex. *Cereb Cortex* 2015; **25**(6)**:** 1454-1468.

7. Duan H, Wearne SL, Rocher AB, Macedo A, Morrison JH, Hof PR. Age-related dendritic and spine changes in corticocortically projecting neurons in macaque monkeys. *Cereb Cortex* 2003; **13**(9)**:** 950-961.

8. Radley JJ, Rocher AB, Rodriguez A, Ehlenberger DB, Dammann M, McEwen BS *et al.* Repeated stress alters dendritic spine morphology in the rat medial prefrontal cortex. *J Comp Neurol* 2008; **507**(1)**:** 1141-1150.

9. Sholl DA. Dendritic organization in the neurons of the visual and motor cortices of the cat. *J Anat* 1953; **87**(4)**:** 387-406.

10. Rochefort NL, Konnerth A. Dendritic spines: from structure to in vivo function. *EMBO Rep* 2012; **13**(8)**:** 699-708.

11. Rodriguez A, Ehlenberger DB, Dickstein DL, Hof PR, Wearne SL. Automated three-dimensional detection and shape classification of dendritic spines from fluorescence microscopy images. *PLoS One* 2008; **3**(4)**:** e1997.

12. Torchiano M. Effsize - a package for efficient effect size computation, R Package version 0.7.1. 2016, 10.5281/zenodo.196082.

13. Cohen J. A power primer. *Psychol Bull* 1992; **112**(1)**:** 155-159.

14. Langmead B, Salzberg SL. Fast gapped-read alignment with Bowtie 2. *Nat Methods* 2012; **9**(4)**:** 357-359.

15. Anders S, Pyl PT, Huber W. HTSeq--a Python framework to work with high-throughput sequencing data. *Bioinformatics* 2015; **31**(2)**:** 166-169.

16. Durinck S, Spellman PT, Birney E, Huber W. Mapping identifiers for the integration of genomic datasets with the R/Bioconductor package biomaRt. *Nat Protoc* 2009; **4**(8)**:** 1184-1191.

17. Law CW, Chen Y, Shi W, Smyth GK. voom: Precision weights unlock linear model analysis tools for RNA-seq read counts. *Genome Biol* 2014; **15**(2)**:** R29.

18. Eden E, Navon R, Steinfeld I, Lipson D, Yakhini Z. GOrilla: a tool for discovery and visualization of enriched GO terms in ranked gene lists. *BMC Bioinformatics* 2009; **10:** 48.

19. Chen EY, Tan CM, Kou Y, Duan Q, Wang Z, Meirelles GV *et al.* Enrichr: interactive and collaborative HTML5 gene list enrichment analysis tool. *BMC Bioinformatics* 2013; **14:** 128.

20. Zhang Y, Chen K, Sloan SA, Bennett ML, Scholze AR, O'Keeffe S *et al.* An RNA-sequencing transcriptome and splicing database of glia, neurons, and vascular cells of the cerebral cortex. *J Neurosci* 2014; **34**(36)**:** 11929-11947.

21. Shen L. GeneOverlap: Test and visualize gene overlaps, 2013, <http://shenlab-sinai.github.io/shenlab-sinai/>.

22. Newman AM, Liu CL, Green MR, Gentles AJ, Feng W, Xu Y *et al.* Robust enumeration of cell subsets from tissue expression profiles. *Nat Methods* 2015; **12**(5)**:** 453-457.

23. Kuhn A, Thu D, Waldvogel HJ, Faull RL, Luthi-Carter R. Population-specific expression analysis (PSEA) reveals molecular changes in diseased brain. *Nat Methods* 2011; **8**(11)**:** 945-947.

24. Gautier L, Cope L, Bolstad BM, Irizarry RA. affy--analysis of Affymetrix GeneChip data at the probe level. *Bioinformatics* 2004; **20**(3)**:** 307-315.

25. Gentleman R. annotate: Annotation for microarrays, R package version 1.52.1. 2016, 10.18129/B9.bioc.annotate.

26. Krijthe JH. Rtsne: T-Distributed Stochastic Neighbor Embedding using a Barnes-Hut Implementation, 2015, <https://github.com/jkrijthe/Rtsne>.

27. Fromer M, Roussos P, Sieberts SK, Johnson JS, Kavanagh DH, Perumal TM *et al.* Gene expression elucidates functional impact of polygenic risk for schizophrenia. *Nat Neurosci* 2016; **19**(11)**:** 1442-1453.

# SUPPLEMENTARY FIGURES

## Supplementary Figure 1. Analysis of dendritic length of apical and basal trees of reconstructed prelimbic cortex layer III pyramidal neurons.


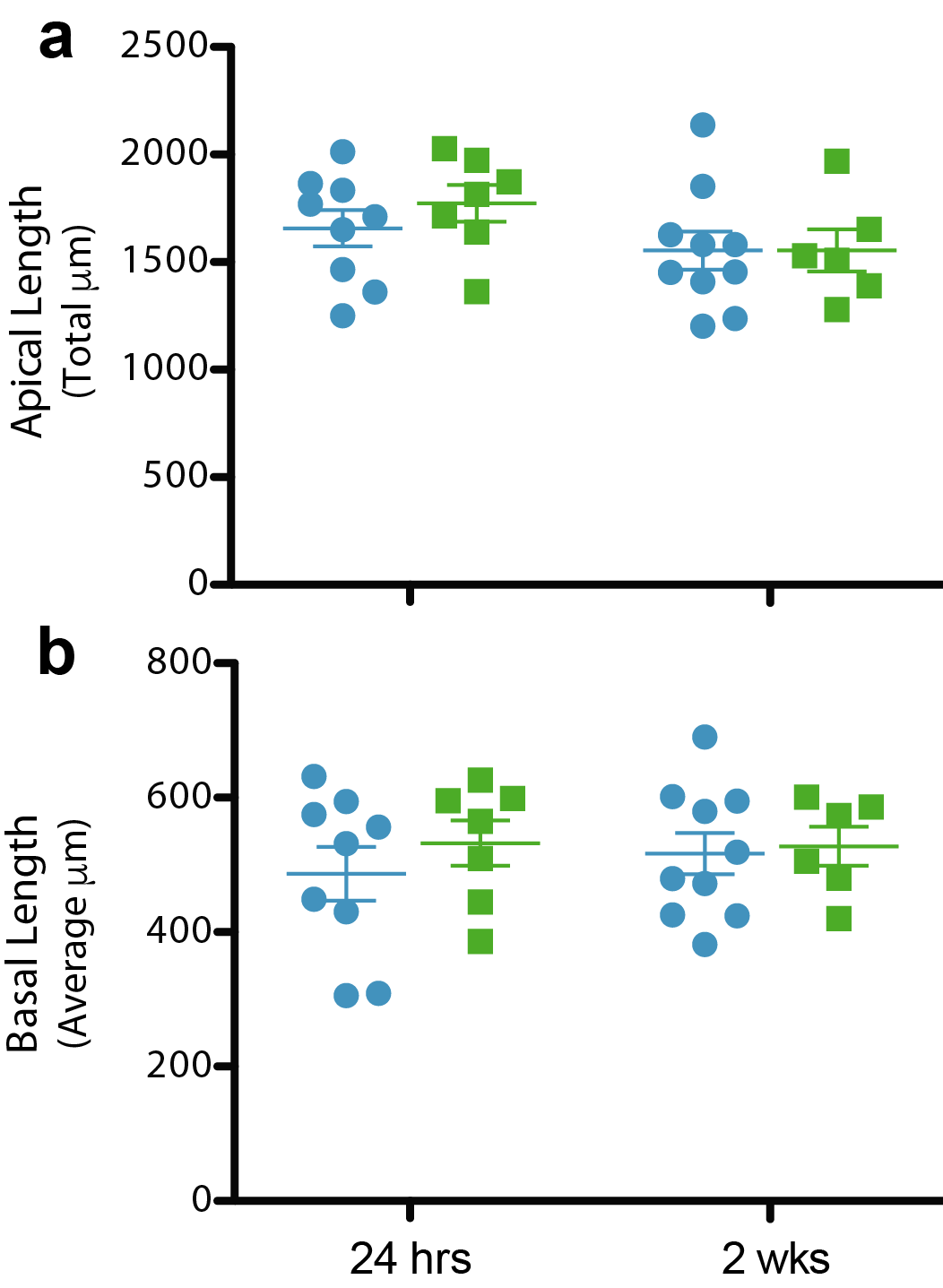


**(a-b)** No differences in total length of apical dendritic trees (a) or average length of basal dendritic trees (b) between treatments and developmental time points.

## Supplementary Figure 2. *In silico* cytometry of microdissected cells to predict source material.


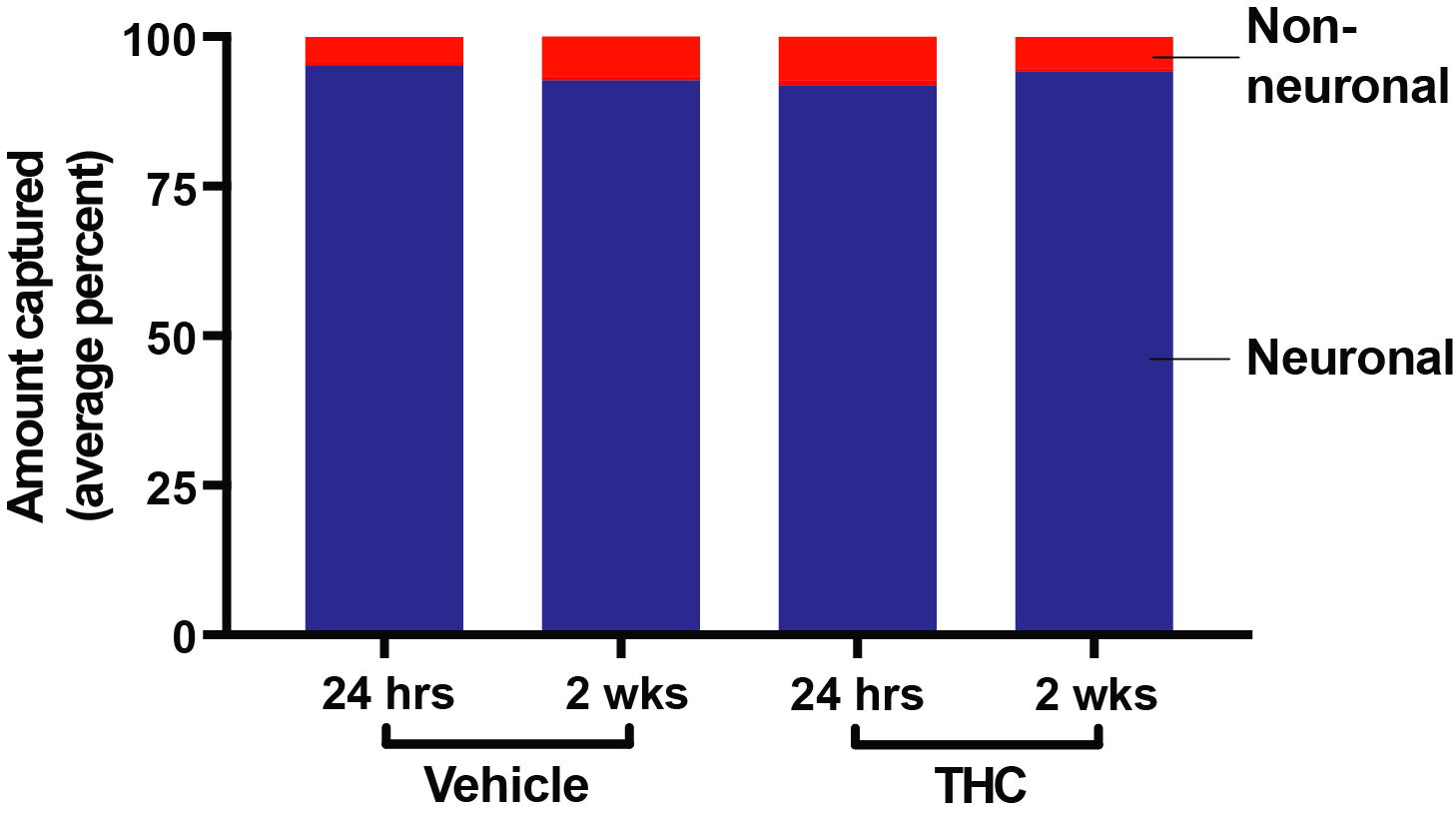


After capturing pyramidal neurons from layer III PrL cortex of vehicle- or THC-treated, *in silico* cytometry of each animal revealed that the microdissected material derived predominantly from neurons, with no statistical differences in relative neuronal contribution across the treatment and developmental groups.

## Supplementary Figure 3. Ontological assessment of differentially expressed transcripts at 24 hours or 2 weeks after adolescent THC exposure.


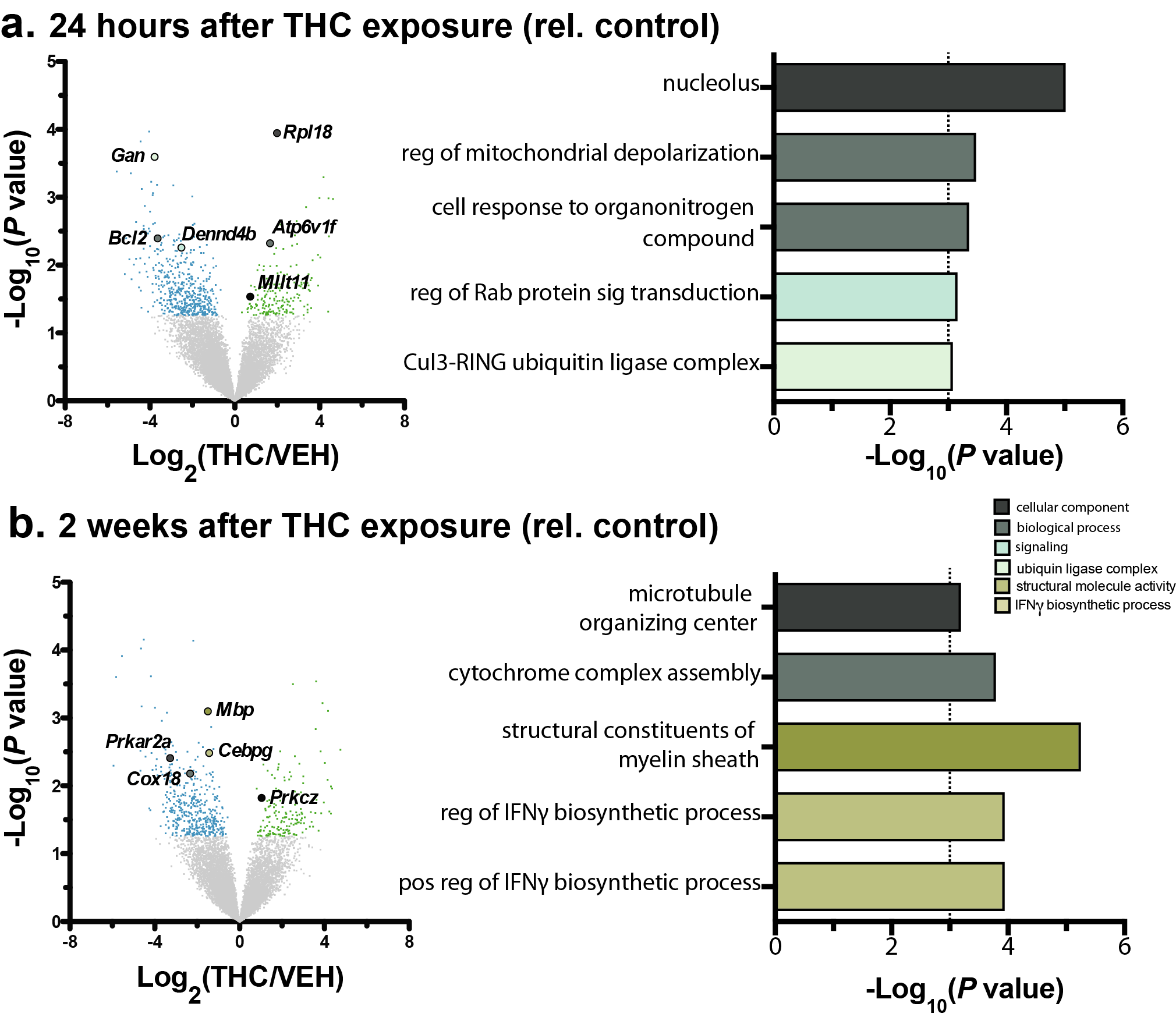


**(a-b)** Ontological enrichment of differentially expressed genes induced 24 hours (a) and 2 weeks (b) after THC exposure. Vertical lines indicate *P*<0.001 (right panels).

## Supplementary Figure 4. Biological processes engaged by normal development in VEH-treated controls.


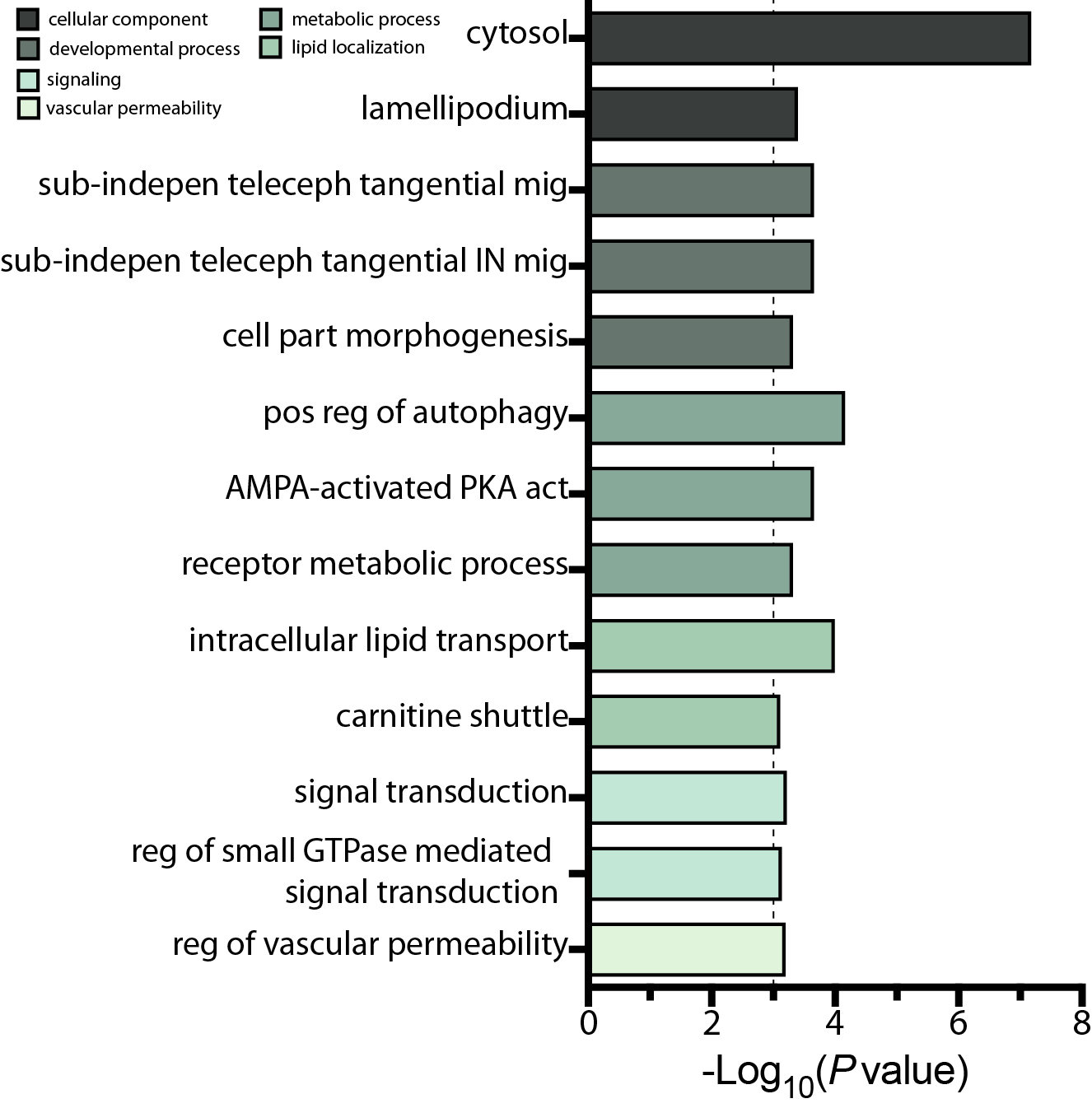


Genes differentially expressed by developmental in vehicle-treated controls engaged biological processes related to development, metabolism and signaling, as well as pathways related to cytosolic components, but not chromatin organization.

## Supplementary Figure 5. Differentially expressed genes identified by RNA-sequencing were replicated using NanoString.


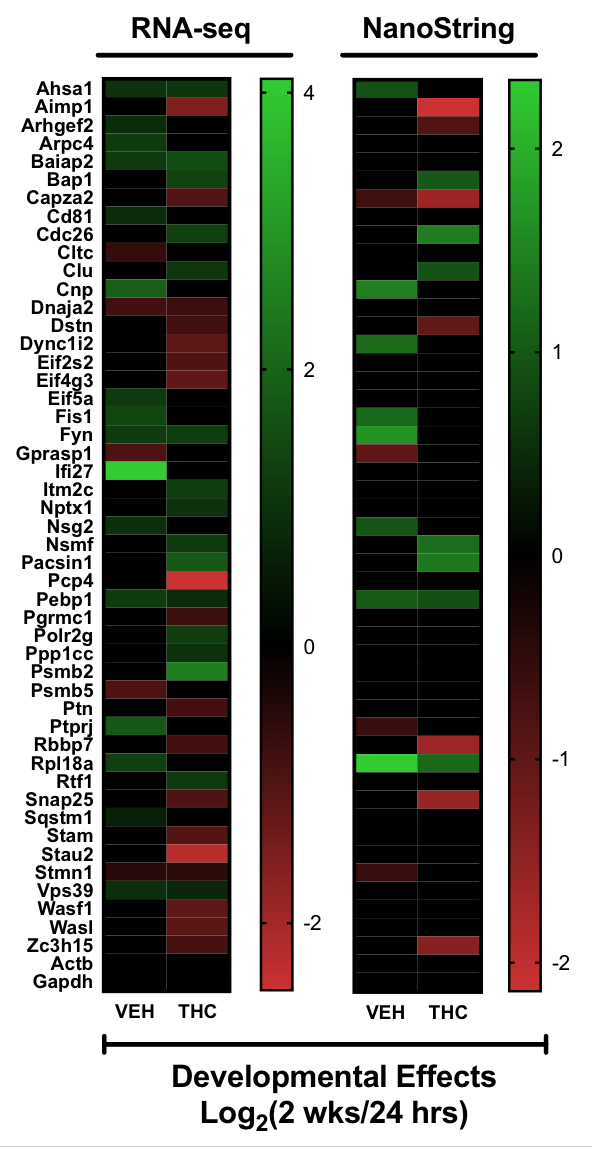


Differentially expressed genes—selected for validation based on their abundance, fold change and association with dysregulated pathways—were measured using NanoString and there was prominent concordance between the sequencing results and NanoString. Only statistically significant differences are colored and scaled to log_2_(ratio).

## Supplementary Figure 6. Enrichment analysis of red, turquoise, cyan and blue schizophrenia CommonMind Consortium (CMC) modules.


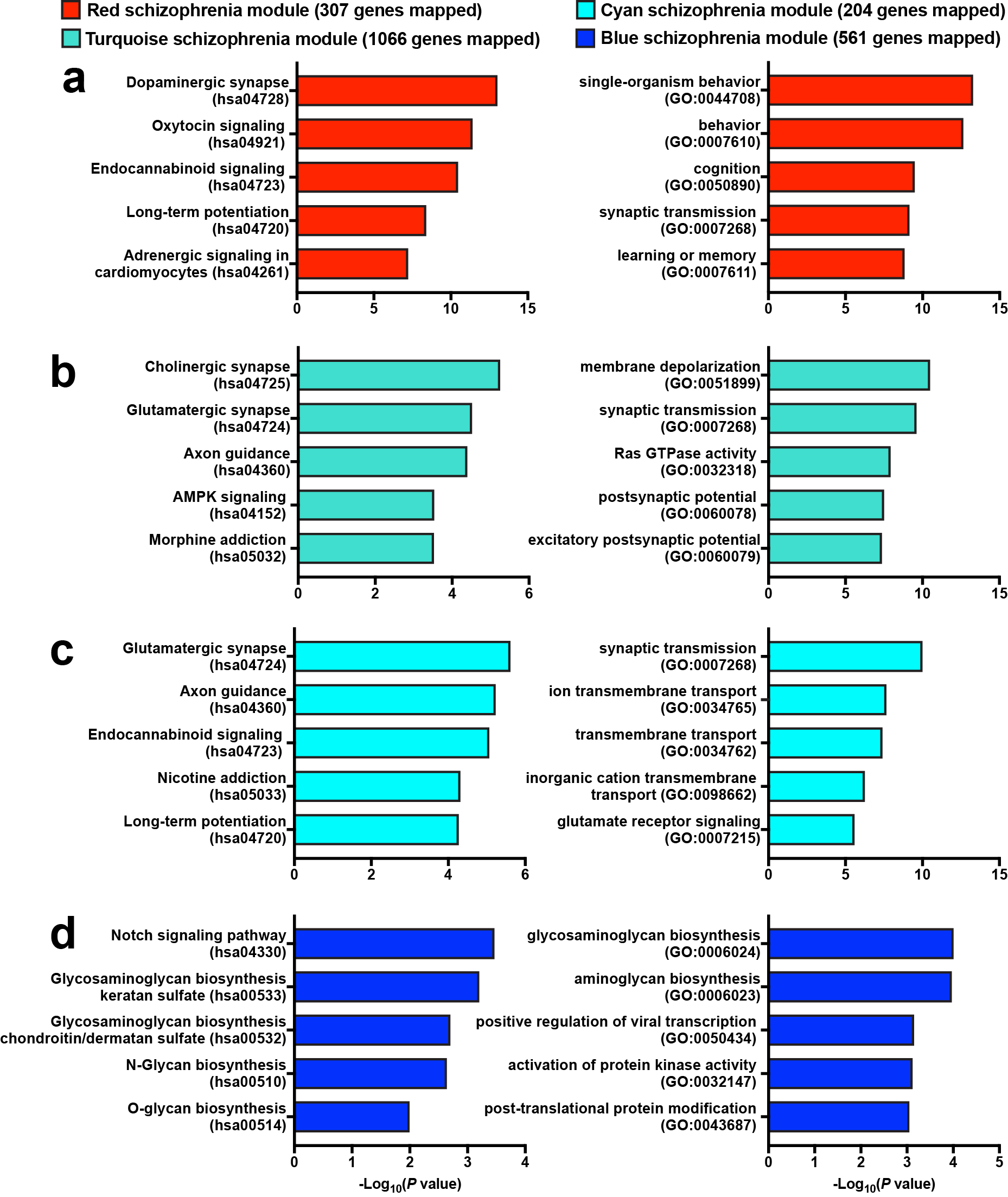


**(a-d)** Enrichment analysis was performed on genes coexpressed in subjects with schizophrenia making up the red (a), turquoise (b), cyan (c), and blue (d) schizophrenia CMC modules. The red, turquoise and cyan modules in particular, which overlapped with developmentally dysregulated and developmentally coexpressed genes, relate to synaptic plasticity and endocannabinoid signaling. Left = KEGG 2016 pathways, right = GO biological processes.

## Supplementary Figure 7. Network of genes, coexpressed in the context of developmental THC and schizophrenia, are predicted to impact cytoskeletal regulation and neurite branching.


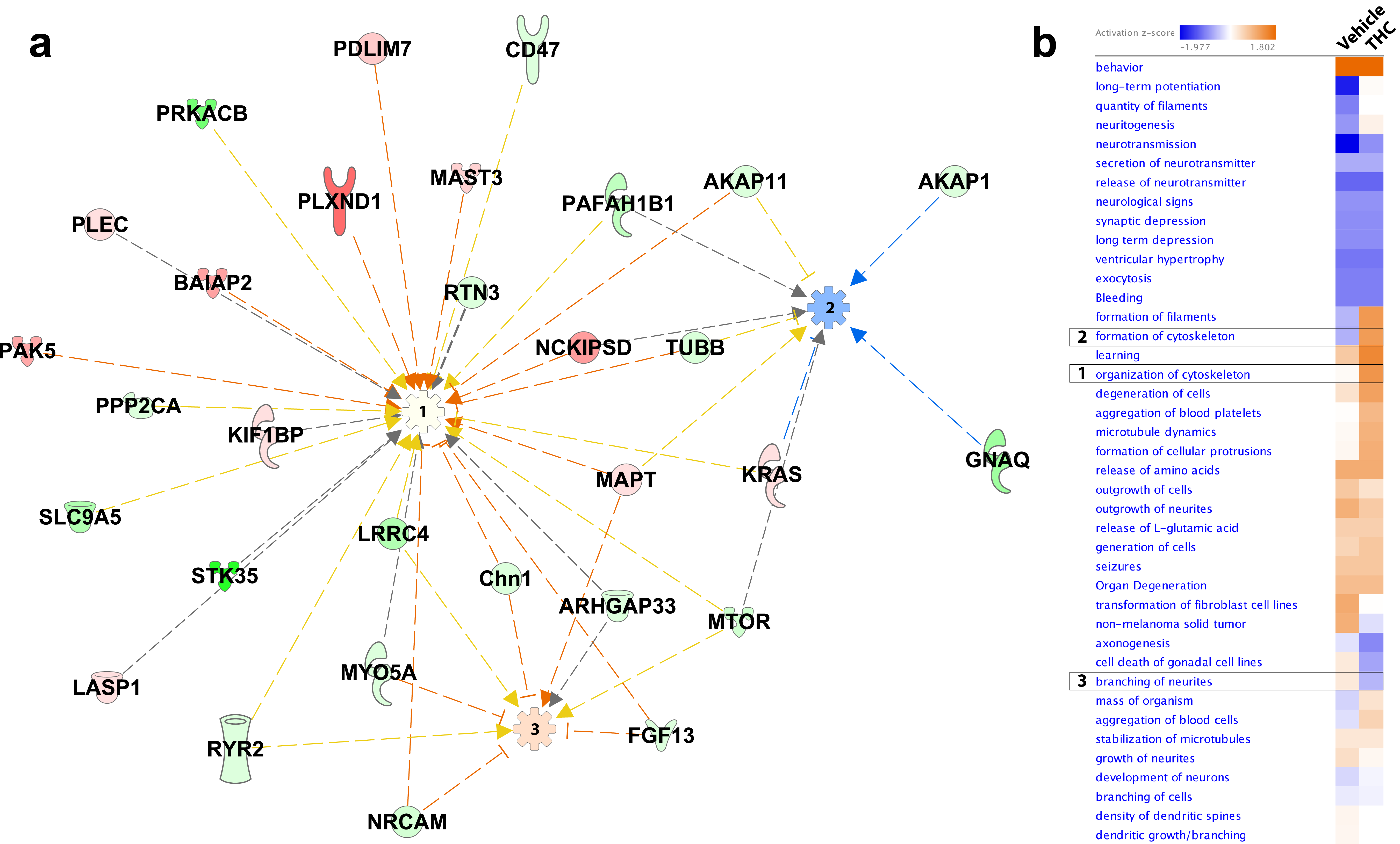


**(a)** Pathway analysis on 181 genes in vehicle animals showing cytoskeletal organization (Hub #1), cytoskeletal formation (Hub #2), and neurite branching (Hub #3). For network and predicted outcomes in THC animals, see Figure 5. **(b)** Heat map showing activity scores between vehicle- and THC-treated animals based on the differential expression pattern of these genes.

# SUPPLEMENTARY TABLES

## Supplementary Table 1. (a) Differential gene expression of Nissl-stained and microdissected pyramidal and non-pyramidal populations from layer III prelimbic (PrL) cortex. (b) Comparison between pyramidal enrichment and neuronal enrichment used for regression analysis.

## Supplementary Table 2. Differential gene expression of pyramidal neurons from layer III prelimbic cortex in animals exposed to either THC or vehicle during adolescence.

**Supplementary Table 3.** Validation of RNA-sequencing data using 50-gene custom NanoString gene expression panel.

## Supplementary Table 6. Overlap analysis (odds ratio, OR) between genes coexpressed in schizophrenia (schizophrenia CMC modules across the columns) and differentially- or co-expressed genes after developmental THC (developmental THC modules down the rows). **P*<0.05, ***P*<0.01, ****P*<0.001.

## Supplementary Table 4. In animals with adolescent THC exposure, developmentally dysregulated genes significantly associated with histone and chromatin modification.

| GO Term | Differentially expressed genes |
| --- | --- |
| histone modification | HDAC5, ENY2, PAXIP1, LDB1, SETD7, RNF8, ARID4A, RTF1, HDAC6, RBBP5, SMYD2, MEN1, NAA50, PRMT5, HIPK4, MGEA5, BRD1, RBM14, KDM4C, BRPF1, MCRS1, USP22, USP3, SETD1A, SETMAR, SIRT6, SIRT3, KAT2A, KANSL3, WAC, ATM, BCOR, BAP1, EZH2, CUL4B |
| chromatin modification | HDAC5, ENY2, PAXIP1, L3MBTL2, LDB1, SETD7, RNF8, ARID4A, RTF1, HDAC6, BAG6, ING2, RBBP5, SMYD2, RBBP7, MEN1, NAA50, HIPK4, MGEA5, PRMT5, SMARCC1, RBM14, BRD1, KDM4C, BRPF1, MCRS1, USP22, USP3, SETD1A, SETMAR, SIRT6, ARID1A, SIRT3, KAT2A, KANSL3, WAC, ATM, BCOR, ITGB3BP, BAP1, CUL4B, EZH2 |

## Supplementary Table 5. Genes from the greenyellow coexpression module (based on WGCNA) significantly associated with organelle and cellular component organization, and histone and chromatin modification.

| GO Term | Coexpressed genes |
| --- | --- |
| organelle organization | MLL1 (HRX), Histone deacetylase class II, HDAC6, NIP3, FUNDC1, MRPL38, ATF/CREB, UBPY, TRAPPC6A, TMEM48, Galpha(i)-specific amine GPCRs, YARS2, Ccdc56, PHF15, C/EBP, C/EBPgamma, ANP32B (april), MAP1S, Stathmin, GORASP1(GRASP65), CDC26, GOLPH3, SKP1, BS69, C14orf169, SAS10, RBBP7 (RbAp46), Dynlrb2, DYNLRB, WASF1(WAVE1), CD24, POR1, LZTS2, VPS16, TOP1MT, PP5, CLASP2, MICAL2, APXL, FLII, TAO2, Rab-3, FIP200, C6orf182, PI3K class II, PI3K class II (CII-alpha), NKD2, MSP58, PYGO1, CACNA1 L-type, PI4KII, AP-1, c-Jun, c-Jun/c-Jun, hCAP-H2, CAP-H/H2, Pleckstrin, DMAP1, SEC22A, TTC19, PRMT5, MUL, ING4, PKC-zeta, PKC, RNF8, SSSCA1, AMPK alpha 1 subunit, AMPK alpha subunit, DYNLT, Dynein, axonemal, light chains, Tctex-1, Casein kinase I, MLH1, DNAJB6 (Hdj-1), VDAC 1, Clusterin, ZPR1, Syntaxin 3, RanBPM, SEC16A, DDB2, Sirtuin, Sirtuin6, FEN1, BAP1, HCF1, GOS-28, YME1L1, Annexin II, CABIN1, LDB1, FTS, PEX13, KLHL13, Tubulin alpha, Tubulin alpha 8, BET1, TFIIH p52 subunit, PMP70, Dynein 1, cytoplasmic, intermediate chains, DYI2, BAF47 |
| cellular component organization | MLL1 (HRX), Histone deacetylase class II, HDAC6, NIP3, FUNDC1, TORC1, TOR2A, MRPL38, ATF/CREB, UBPY, TRAPPC6A, PhLP, TMEM48, PLSCR3, Serotonin receptor, Galpha(i)-specific amine GPCRs, YARS2, SHC3, SLC39A3 (ZIP3), Ccdc56, GSPT2, OST48, CAPZA2, CAPZA, PHF15, C/EBP, C/EBPgamma, ANP32B (april), MAP1S, Stathmin, RBM9, GORASP1(GRASP65), RPLP1, CDC26, GOLPH3, SKP1, PSMB2, BS69, NF-I, NFIB, C14orf169, RFTN2, SAS10, RBBP7 (RbAp46), Dynlrb2, DYNLRB, WASF1(WAVE1), CD24, POR1, LRFN4, LZTS2, VPS16, TOP1MT, PP5, SEMACAP3, PSMC3, RBM22, CLASP2, MICAL2, APXL, Trichoplein, FLII, PRICKLE2, TAO2, Rab-3, FIP200, SNIP, FAM103A1, C6orf182, PI3K class II, PI3K class II (CII-alpha), NKD2, MSP58, PYGO1, CACNA1D, CACNA1 L-type, eIF3S10, PI4KII, AP-1, c-Jun, c-Jun/c-Jun, hCAP-H2, CAP-H/H2, Pleckstrin, DMAP1, SEC22A, TTC19, PRMT5, MUL, eIF2C1 (Argonaute-1), ING4, PKC-zeta, PKC, RNF8, SSSCA1, TARBP2, Tetraspanin-2, AMPK alpha 1 subunit, AMPK alpha subunit, DYNLT, Dynein, axonemal, light chains, Tctex-1, Casein kinase I, MLH1, DNAJB6 (Hdj-1), VDAC 1, Clusterin, ZPR1, CRMP3, Syntaxin 3, RanBPM, SEC16A, DDB2, Sirtuin, Sirtuin6, GFOD2, FEN1, BAP1, HCF1, KEAP1, GOS-28, YME1L1, Annexin II, RPS12, CABIN1, LDB1, SFRS1 (SF2), FTS, PEX13, TBCC, KLHL13, Kv9.1, Semaphorin 4D, Tubulin alpha, Tubulin alpha 8, Hap-1, BET1, TFIIH p52 subunit, TREM2, KCTD10, FLJ22875, PMP70, Dynein 1, cytoplasmic, intermediate chains, DYI2, BAF47, Galpha(i)-specific metabotropic glutamate GPCRs |
| chromatin modification | MLL1 (HRX), Histone deacetylase class II, HDAC6, NIP3, ATF/CREB, PHF15, C/EBP, C/EBPgamma, SKP1, BS69, C14orf169, SAS10, RBBP7 (RbAp46), TOP1MT, PP5, MSP58, AP-1, DMAP1, PRMT5, MUL, ING4, PKC, RNF8, AMPK alpha 1 subunit, AMPK alpha subunit, DDB2, Sirtuin, Sirtuin6, BAP1, HCF1, CABIN1, LDB1, BAF47 |
| histone modification | MLL1 (HRX), Histone deacetylase class II, HDAC6, ATF/CREB, PHF15, SKP1, C14orf169, PP5, MSP58, AP-1, DMAP1, PRMT5, MUL, ING4, PKC, RNF8, AMPK alpha 1 subunit, AMPK alpha subunit, DDB2, Sirtuin, Sirtuin6, BAP1, HCF1, LDB1 |
